# Scientific criticism. A plea for continuous and open peer review

**DOI:** 10.3389/fphys.2025.1661509

**Published:** 2025-09-04

**Authors:** Edward Vigmond, Ruben Coronel

**Affiliations:** ^1^ Lab IMB UMR5251, Université de Bordeaux, Bordeaux, France; ^2^ IHU Liryc, Fondation Bordeaux Université, Pessac, France; ^3^ Department of Experimental Cardiology, Amsterdam University Medical Centers, Amsterdam, Netherlands

**Keywords:** scientific critique, peer review, scientific criticism, review process, publishing

## Introduction

On 1 January 2025 the office of Specialty Chief Editor (SCE) of Frontiers in Cardiac Electrophysiology was transferred to Dr. Edward Vigmond. The Journal is a subsection of Frontiers in Physiology, and welcomes basic science and clinically oriented manuscripts that address arrhythmogenic mechanisms, their modulatory factors (pharmacologic, autonomic, dietary, genetic, etc.), and computer simulations thereof. There is a close rapport with other Specialty sections, outside the field of Physiology (Cardiovascular Medicine - Arrhythmology).

Since the start of the Journal section, it has witnessed a growth both in submitted manuscripts and in the number of Research Topics (special issues). The transfer of the Specialty Chief Editorship to Dr. Vigmond will facilitate an expansion of the Journal content towards papers reporting computer simulation studies related to arrhythmia mechanisms and their modulation. However, many articles cannot be considered solely experimental or solely modelling. Due to biological system complexity, modelling is helpful to interpret results, and, conversely, experiments are needed to build models and validate their predictions. Thus, we also forsee and welcome more hybrid papers, synergistically utilizing the two approaches. The increased use of artificial intelligence is also anticipated in articles, but its role should be to provide insight into mechanisms of cardiac electrophysiology and not be an end unto itself. Finally, we would like to take this opportunity to share our views on the role of peer review in the journal section.

## Present practice of peer review

Frontiers is characterized by high quality, fast and partially open peer review from a global network of reviewers (Ben Messaoud et al., 2023). Partially open implies that the identity of the Review Editors (reviewers) is published with the accepted papers, but that it is not revealed during the review process. During the review process itself, authors and reviewers have an open interaction (Ross-Hellauer, 2017). While we applaud this transparency and recognize that this aspect is one of the attractive features of publishing with Frontiers, we ask ourselves whether the peer review process may not be taken a step further.

## Discussion

In the times when scientific publication solely depended on printed journals, page limitations by necessity required a selection process if the volume of the number of submitted papers exceeded the number of permitted volume (pages). Thus, a limited number of “peers”, usually established scientists working in the same field as the authors of the respective paper, were asked to provide an assessment (the reviewer’s report) of the submitted paper. The editors of the Journal then could use these critiques to grade papers and select those that were to be published, while rejecting others (Europe PMC, 1999).

If a paper was rejected, it was a common practice to take advantage of the generated comments, improve the paper accordingly and send the paper to another journal. In the majority of cases, all papers would be published, although usually in a journal with less stature (quantitatively expressed in the impact factor). Some papers were so much improved that they were published in a journal with an even higher impact factor (Opthof et al., 2000).

The peer review process has its limitations. It is often difficult to find suitable reviewers who are experts on the ensemble of topics and techniques of the submitted paper. Present day scientific papers often entail multiple techniques and multiple areas of expertise. For example, a paper on Brugada Syndrome may have used molecular biology, gene editing, patch clamp methods, cell isolation and clinical electrocardiology. It is almost impossible to find a reviewer knowledgeable on all of these fields. Even two or three reviewers would not be able to cover all aspects. Thus, if a paper is accepted following this incomplete review, a reader is likely to find fault with content that was not covered by the expertise of the two or three peers. This property of present peer review may even be exploited by a “smart” author who submits a paper whose topics not entirely covered by the target journal, thereby increasing its chance for acceptance. Securing reviewers, even on a single topic, may also be difficult due to the limited size of the pool of experts. A situation which is exacerbated by the disincentive of an increased workload for the already busy reviewer, with little benefit (El-Guebaly et al., 2023).

Present publication practice does not depend on printed journals and one may question the selective role of peer review in the era of on-line scientific publication, as is the case with Frontiers.

## Scientific process

Contrary to what is commonly expressed, the publication of a scientific paper is the start, not the end of the scientific process. We all celebrate when a paper that we have co-authored has been accepted to be published in a journal. After acceptance, we generally close the files associated with the paper, while shifting our attention to new projects and papers. However, the mere structure of the published papers suggests otherwise. The Introduction should explain why we were driven to undertake the study, and the Methods section of our paper should allow replication of the procedures or experiments. Although replication studies are not very popular to do and, therefore, are rare, failure to replicate is a major stimulus to refine, improve or correct. In many cases, studies showing a failure to replicate have the words “not” or “controversy” in the title (Opthof et al., 2007; Chung et al., 2021; Baartscheer et al., 2016). These papers are often difficult to publish, because the reviewers are often biased on the subject, and because the controversy may be associated with strong emotions, especially when funding depends on publication success (Coronel, 2020).

## The role of criticism in science

Asking scientific questions is at the core of the scientific process. The ensuing discussion is the only method that we have to improve our understanding, to differentiate between false and true, and to generate new approaches to the problem. Of course, we can anticipate some of the criticism, but this discussion section of a paper should not be restrictive and should only be considered as the “ouverture” of the scientific opera.

It is difficult to ask scientific questions or to criticize the work of a colleague. Young scientists are aware that they are entering a network of scientists, and that critical notes may be interpreted as inappropriate by established scientists who resent their work being questioned. Publications are vitally important for career progress and jobs, so reviewing interests may be conflicted. Yet, criticism is the only method that we have to propagate science, especially in an era when science is considered an opinion. Scientific criticism is not ad hominem but ad rem. It is the exchange of arguments, both positive and negative, based on facts and scientific observations. “And yet it moves”, as expressed by Galilei shows the ultimate form of scientific criticism, even though it was mistaken as an opinion that should be suppressed. Double blinding of the review process tries to remove prejudice of the reviewer (Parmanne et al., 2023), but self references and the unique nature of particular labs reduce its efficacy.

## On-line scientific criticism

Now that scientific journals are not limited by page numbers, the peer review process can be disconnected from the selection process and can be targeted to what it was meant for, scientific criticism. This implies that a high rejection rate is not necessarily a marker for quality. Of course, a first stage of selection is still needed to exclude papers that are outside the scope of the journal, poorly written, do not conform to the ethical requirements, are inadequately formatted, plagiarize, or that show double publication, et cetera. Of particular note are fraudulent papers produced by papermills, increasingly generated by AI. Frontiers has responded by employing AI to cull these papers in an ever-escalating war. After that, the purely scientific process takes place. The reports of the peer reviewers, which should be critiques and not just criticisms, are published, together with the response of the authors. The critiques and the responses are not ad hominem but ad rem, and readers should be able to add their scientific comments, to which authors can add their responses. The journal provides a moderator to remove unscientific content. In this manner, open, permanent and interactive (peer) review will secure scientific criticism (even involving old time papers), and science will benefit.

## References

[B1] Baartscheer A. Schumacher C. A. Wust R. C. Fiolet J. W. Stienen G. J. Coronel R. (2016). Empagliflozin decreases myocardial cytoplasmic Na+ through inhibition of the cardiac Na+/H+ exchanger in rats and rabbits. Diabetologia 60, 568–573. 10.1007/s00125-016-4134-x 27752710 PMC6518059

[B2] Ben Messaoud K. Schroter S. Richards M. Gayet-Ageron A. (2023). Analysis of peer reviewers' response to invitations by gender and geographical region: cohort study of manuscripts reviewed at 21 biomedical journals before and during covid-19 pandemic. BMJ 381, e075719. 10.1136/bmj-2023-075719 37311585 PMC10471900

[B3] Chung Y. J. Park K. C. Tokar S. Eykyn T. R. Fuller W. Pavlovic D. (2021). Off-target effects of sodium-glucose co-transporter 2 blockers: empagliflozin does not inhibit Na+/H+exchanger-1 or lower [Na+]iin the heart. Cardiovasc Res. 117, 2794–2806. 10.1093/cvr/cvaa323 33135077 PMC8683707

[B4] Coronel R. (2020). On publication strategies. Another advice to a beginning scientist. Front. Physiol. 11, 1073. 10.3389/fphys.2020.01073 33013460 PMC7500198

[B5] El-Guebaly N. Foster J. Bahji A. Hellman M. (2023). The critical role of peer reviewers: challenges and future steps. Nord. Alkohol Nark. 40 (1), 14–21. 10.1177/14550725221092862 36793486 PMC9893124

[B6] Europe PMC (1999). Pros and cons of open peer review. Nat. Neurosci. 2 (3), 197–198. 10.1038/6295 10195206

[B7] Opthof T. Furstner F. Van Geer M. Coronel R. (2000). Regrets or no regrets? No regrets! the fate of rejected manuscripts. Cardiovasc Res. 45, 255–258. 10.1016/S0008-6363(99)00339-9 10728343

[B8] Opthof T. Coronel R. Wilms-Schopman F. J. G. Plotnikov A. N. Shlapakova I. N. Danilo P. (2007). Dispersion of repolarization in canine ventricle and the electrocardiographic T wave: Tp-e interval does not reflect transmural dispersion. Heart Rhythm 4, 341–348. 10.1016/j.hrthm.2006.11.022 17341400

[B9] Parmanne P. Laajava J. Järvinen N. Harju T. Marttunen M. Saloheimo P. (2023). Peer reviewers’ willingness to review, their recommendations and quality of reviews after the Finnish medical journal switched from single-blind to double-blind peer review. Res. Integr. Peer Rev. 8 (1), 14. 10.1186/s41073-023-00140-6 37876004 PMC10598992

[B10] Ross-Hellauer T. (2017). What is open peer review? A systematic review. F1000Research 6, 588. 10.12688/f1000research.11369.2 28580134 PMC5437951

